# (-)-Epigallocatechin-3-O-Gallate Regulates Muscle Growth, Antioxidant Status, and Nutritional Composition of Juvenile Common Carp (*Cyprinus carpio* L.)

**DOI:** 10.1155/2024/7134404

**Published:** 2024-03-20

**Authors:** Jiali Mi, Dan Liu, Shaoyang Zhi, Xiao Yan, Chaobin Qin, Xinxin Xu, Luming Wang, Guoxing Nie

**Affiliations:** ^1^Aquatic Animal Nutrition and Feed Research Team, College of Fisheries, Henan Normal University, Xinxiang 453007, China; ^2^College of Life Science, Henan Normal University, Xinxiang 453007, China

## Abstract

Among the polyphenolic compounds commonly found in green tea, (-)-epigallocatechin-3-gallate (EGCG) is known to have various functions, including the promotion of antioxidant and myofiber growth in mammals. However, the effect of EGCG on common carp (*Cyprinus carpio* L.) remains poorly understood. To evaluate the role of EGCG on juvenile common carp, 255 fish (initial weight 17.33 ± 0.34 g) were fed with five experimental diets containing 0, 0.05, 0.25, 0.5, and 1 g/kg EGCG. The results showed that diet supplementation with 0.05–0.5 g/kg EGCG supplementation significantly enhanced the specific growth rate (SGR) and reduced the feed conversion ratio (FCR). Weight gain rate (WGR) was significantly enhanced in the 0.25 and 0.5 g/kg EGCG groups. As for antioxidants, 0.25–1 g/kg EGCG significantly reduced protein carbonyl (PC) content and upregulated superoxide dismutase (*sod*) gene expression in the muscle. As for muscle nutritional composition, 0.05–0.5 g/kg EGCG increased total amino acid (TAA) and flavor amino acid (FAA) contents, likely via the rapamycin (mTOR) signaling pathway. Muscle n-3 polyunsaturated fatty acids (n-3 PUFA) content was increased with 0.5 g/kg EGCG, presumably owing to the upregulation of fatty acyl elongase 5 (*elovl5*), long-chain fatty acyl-CoA synthetase 6 (*acsl6*), and peroxisome proliferator-actiated receptor *α* (*pparα*). Dietary EGCG (0.05–1 g/kg) significantly increased muscle hardness and chewiness, accompanied by an increase in myofiber density. EGCG supplementation (0.25–1 g/kg) increased the pH value and reduced lactate contents in the muscle. However, muscle crude lipid and hydroxyproline contents significantly decreased with 1 g/kg EGCG. Overall, quadratic regression analysis of WGR, SGR, TAA, and FAA showed that optimal EGCG (0.46–0.52 g/kg) dietary supplementation improved the growth and nutritional composition of juvenile common carp.

## 1. Introduction

Aquaculture plays a crucial role in providing high-quality protein to the human diet, and the global demand for fish is on the rise [1]. Several intensive aquaculture techniques have been used to increase fish production. However, as market demand for high-quality meat products continues to increase, there is an increasing focus on aquatic products quality [2]. Nutrition, muscle fiber structure, sensory quality, flavor, and physical properties are factors used to evaluate fish muscle quality [3]. Unfortunately, high-density culture induces a strong stress response that leads to the deterioration of fish muscle quality, such as lipid and protein oxidation, muscle fiber breakage, texture softening, and reduced water-holding capacity [4, 5]. The occurrence of oxidative stress in muscles often results in excessive production of malondialdehyde (MDA) and protein carbonyl (PC), which can lead to fish spoilage and deterioration. Furthermore, these processes can negatively affect human health [5]. In addition, the flavor of fish plays a crucial role in consumer acceptance, especially for freshwater fish, as an overly soft, muscular texture is often undesirable. The characteristics of muscle texture are usually determined by a combination of factors such as muscle fiber development, collagen content, and muscle fat content. In previous studies, *Eucommia ulmoides* extract [6] and tea polyphenols [7] improve the muscle collagen content of grass carp (*Ctenopharyngodon idellus*). Moreover, GIFT tilapia (*Oreochromis mossambicus*) fed tea polyphenols [8] and Nile tilapia (*Oreochromis niloticus*) fed grape seed proanthocyanidin extract [9] and cinnamaldehyde [10] showed improved antioxidant capacity, muscle fiber properties, and muscle hardness.

Green tea (*Camellia sinensis*) is rich in polyphenols, particularly catechins (flavonoids). Extensive research has shown that tea polyphenols enhance the sensory qualities of muscles. Moreover, numerous studies have established that dietary tea polyphenols have a positive effect on various aspects such as growth performance, lipid metabolism, flesh quality, and stress resistance in multiple fish larvae [7, 8, 11–14]. Among several catechin monomers, (-)-epigallocatechin-3-gallate (EGCG) accounts for the highest proportion, generally ranging from 30% to 50%. Initially, EGCG gained popularity in the food industry because of its strong antioxidant ability. A previous study highlighted the high antioxidant capacity of EGCG, which effectively helps resist stress-induced cell damage [15]. Previous studies have suggested that EGCG plays various biological functions, including the enhancement of the immune defense response [16–19], promoting osteoblast differentiation, and preventing muscle atrophy in vivo and in vitro [20]. However, there are few reports on the effects of EGCG on muscle growth and fish quality. Our previous studies showed that (-)-epicatechin (EC), another catechin compound, enhanced muscle antioxidant capacity and myofiber density [21]. Given the strong antioxidant capacity of EGCG, we hypothesized that adding EGCG to the diet might improve fish flesh quality, particularly in terms of nutritional quality and physicochemical properties.

Common carp (*Cyprinus carpio* L.) is a freshwater fish popularly consumed in China because of its nutritional and cultural value. The muscles of the common carp have abundant plentiful fatty acids, proteins, and minerals [22]. However, recently, the nutritional value of common carp muscles has deteriorated owing to intensive culture and environmental pollution [23, 24]. This study is the first to focus on the effects of dietary EGCG supplementation on the antioxidant capacity, nutritional composition, and textural quality of muscles in juvenile common carp. Our aim is to provide a solid foundation for improving muscle nutritional value and antioxidant capacity in common commercial carp.

## 2. Materials and Methods

### 2.1. Experimental Diets and Design

Five test diets were formulated with the same levels of protein and lipid but with varying concentrations of EGCG (0 (CN), 0.05, 0.25, 0.5, and 1 g/kg); the values measured by high-performance liquid chromatography (HPLC) are listed in *Supplementary 1*. The EGCG dose was based on previous studies [7, 8, 11, 18]. The EGCG used in this study was purchased from Nanjing Daosifu Biotech Company (Nanjing, China) and had a purity of 98%. Fully mixed with all ingredients, EGCG was dissolved in water, evenly sprayed on the raw materials, and mixed step-by-step to ensure a uniform content of EGCG in all the test diets. The mixed ingredients were then conveyed to a pellet machine (F-26, Guangzhou Huagong Optical Mechanical & Electrical Technology CO., Ltd.) and cold-extruded with a 2 mm diameter die. Cooling was performed during this process. After drying, the prepared feed is placed in a vacuum-light-proof self-sealing bag and stored at −20°C until use. Common carp were purchased from the Henan Fisheries Research Institute (Zhengzhou, China). The fish were domesticated for two weeks, and then 255 of them (initial weight 17.33 ± 0.34 g) were selected and randomly distributed into 15 tanks (300 L of water and 17 fish per tank) connected to a closed water and oxygen auto-supplemented recirculating aquatic system with three layers of filtration equipment in the greenhouse. Before the formal experiment, carp were fed the control group diets for one week. During the experiment, the circulation pool was cleaned daily, and the water change in the breeding bucket was 1/3 of the total volume. Carp were manually fed three times a day, at 8:00, 12:00, and 16:00 hr for 2 months. The feeding amount was calculated as 3% of the fish body weight and was increased weekly according to the latest weight, which was estimated according to the feed coefficient from a previous similar study [25]. The tanks were supplied with a continuous flow of water, maintaining a dissolved oxygen concentration of 7.0 ± 0.2 mg/L. The water temperature was kept at 26 ± 3°C. The water exchange rate for each tank was maintained at 6.94 L/min (power pump 10,000 L/h). The pH of water was kept at 7.0 ± 1.0.

### 2.2. Sample Collection

After a 2-month period, all fish were individually counted in each tank following a 24 hr fasting period. Prior to sampling, the carp were transferred to a solution containing 60 mg/L tricaine methane sulfonate (MS-222, Sigma) and anesthetized until the fish stopped swimming but were still breathing. All carps were weighed under anesthesia. The carp were then quickly killed by snipping the backbone after anesthesia and dissected from the anus to the throat on ice using sterile surgical tools to remove the internal organs. Subsequently, three carp were randomly selected from each tank, and their morphological indexes were measured. Following careful descaling, white muscle samples were extracted from the dorsal area of the carp (three fish per tank) and preserved in a 4% paraformaldehyde solution for histomorphological (one fish per tank) and immunofluorescent analysis (two fish per tank). Three fish per tank were rapidly dissected, and the removed muscle samples were rapidly placed in liquid nitrogen for enzyme activity assay, lactate content, and real-time quantitative PCR (RT-qPCR) analysis. Dorsal fillets (1 × 1 × 0.5 cm) from both sides of the carp (three fish per tank) were removed for texture analysis. Six fish were collected from each tank, and muscle samples were mixed to determine the proximate compositions and amino acid and fatty acid contents of the fish. Two fish were collected from each tank to determine muscle pH, hydroxyproline content, cooking loss, and drip loss.

### 2.3. Antioxidant Enzyme Activity Analysis

Muscle enzyme activity was measured using a commercial kit following the manufacturer's instructions. Optical density (OD) was measured using a universal microplate reader (AU-5800, Beckman). Muscle samples were homogenized with 9× saline and centrifuged at 4°C (1,789× *g*) for 10 min. The antioxidant indices of the supernatants were determined.

### 2.4. Muscle Proximate Compositions and Physicochemical Properties

Muscle and diet proximate compositions were determined according to the standard methods of the AOAC [26]. Briefly, diet samples were dried at 105°C for at least 4 hr, and the muscle samples were dehydrated by vacuum freeze-drying until reaching a constant weight; then, the moisture content was ascertained. The ash content was ascertained by placing the samples into a muffle furnace at 500°C for 5 hr. Crude protein content was determined by the Kjeldahl method, and crude lipid content was determined by the Soxhlet method (Agilent Technologies, Santa Clara, CA, USA) after petroleum ether extraction. The hydroxyproline content of the muscle was measured using a biochemical kit from Nanjing Jian Cheng.

Fresh muscle samples (1 g) were homogenized, and 9 mL of distilled water was added to determine the pH of the solution using a calibrated pH meter (PHSJ-3F, Shanghai, China). To calculate the cooking loss, a fish flesh sample (approximately 1 g) was packed in a sealed polybag and immersed in water at 80°C for 20 min. In turn, to calculate the dripping loss, the muscle sample (approximately 1 g) was placed on filter paper (constant weight) in a centrifuge tube (50 mL) and centrifuged for 10 min at 500× *g*. Finally, the dripping loss was calculated based on the weight of material absorbed by the filter paper.

### 2.5. Fillet Texture and Histomorphology Analysis

Nine fish were anesthetized in each tank. Dorsal fillets were sliced on both sides. The samples were tested for12 hr using a texture analyzer (TA. XT plus, UK). The detection indicators included fillet hardness, chewiness, springiness, and shear force. Three carp from each group were selected for histomorphological observations. The muscle tissues were stored in 4% paraformaldehyde and then processed through dehydration, transparency, embedding, and cutting into 6 *μ*m thick sections for HE staining using a kit (Solarbio, Beijing). A microscope (Zeiss, Germany) was used to observe and determine the diameter and density of the myofibers according to a previously described method [27].

### 2.6. Muscle Immunofluorescence

Muscle immunofluorescence was assessed according to the methods of Yun et al. [28] and Song et al. [25], with slight adjustments. After dewaxing and rehydration, the slices were incubated with enhanced endogenous peroxidase-blocking buffer (Beyotime) for 10 min to block endogenous catalase activity. Subsequently, the slices were treated with goat serum (Solarbio, Beijing) and incubated with the primary antibodies, including myogenic differentiation antigen (MyoD), paired box 7 (Pax7), myogenin, and myogenic factor 4 (Mrf4) at a dilution of 1 : 1,000 at 4°C. After 12 hr, the slices were incubated with goat anti-rabbit IgG (Alexa Fluor 488, dilution 1 : 1,000) for 1 hr in the dark. Then, the slices were incubated with antifade mounting medium containing 4′, 6-diamidino-2-phenylindole (DAPI) (Beyotime) for 5 min. Prior to each treatment, the slices were washed with PBS. Finally, images were captured using a fluorescence microscope (Zeiss, Germany) and analyzed using Image J with data collected from six independent visual fields.

### 2.7. Amino Acid Contents and Fatty Acid Composition

A 100 mg lyophilized muscle sample was dissolved in 0.1 N HCl (6 mL) in an oven at 110°C for 24 hr. The solution was then filtered and fixed to 50 mL using ultrapure water. Subsequently, the diluted hydrolysate (1 mL) was aspirated for deacidification. After deacidification, the bottom precipitate was dissolved in 1 mL of the diluted sample. Finally, the solution was filtered through a filter membrane (0.22 *μ*m). The final hydrolysate was analyzed using an automatic amino acid analyzer (A300, Germany). Muscle fatty acid content was determined by gas chromatography (Agilent 7890A) as described by Zhang et al. [29], with a few modifications. Briefly, total lipids in freeze-dried samples (100 mg) were extracted in 8 mL of a 2 : 1 volume ratio mixture of twice as much chloroform and methanol. Chloroform and methanol were separated by centrifugation with CaCl_2_ solution; the lower layer solution was dried with nitrogen gas; and the fatty acids were methyl esterified using a mixture of methanol and sulfuric acid at 80°C for 60 min. Finally, the solution was added to n-hexane and passed through a filter membrane (0.22 *μ*m). The relative fatty acid content of the samples was calculated.

### 2.8. qPCR Analysis

Approximately 100–200 mg samples of frozen (−80°C) white muscle were added with TRIzol reagent (Takara, Japan) for total RNA extraction. The extracted RNA was detected by agarose gel electrophoresis, and the concentration was determined by spectrophotometry (Nanodrop 2000 C). RNA was reverse-transcribed into cDNA with a 20 *μ*L volume using the Primecript™ RT reagent kit (Takara). The 2^−*ΔΔCt*^ method was used to determine gene relative-expression level [30]. Primers were tested for amplification efficiency (90%–105%), and the specific sequences and values are shown in *Supplementary 2*. The 18 s ribosomal RNA (18 s) was used as a housekeeping gene [31, 32]. The mRNA levels were detected using the ChamQ Universal SYBP qPCR Master Mix (Vazyme, Nanjing, China) and a LightCycler 480 instrument (Roche, Switzerland). Amplification was performed using 284-well plate amplification in a reaction volume of 10 *μ*L, reaction volume of 5 *μ*L 2 × ChamQ universal-sal SYBP qPCR Master Mix, forward primer and reverse primer 0.3 *μ*L each, template cDNA 0.1 *μ*L, and RNase-free water 4.3 *μ*L. The procedure for quantitative PCR was conducted as follows: 95°C for 3 min, followed by 40 cycles of 95°C for 5 s, and 60°C for 30 s.

### 2.9. Statistical Analysis

All data were tested for equality of variance using Levene's test and analyzed using SPSS 23.0 (IBM Corp., Chicago, USA). Duncan's one-way analysis of variance was used to analyze differences among groups. Data are expressed as means ± standard error, and significance was denoted as *P* < 0.05. The polynomial contrast method was used to analyze linear and quadratic data. The optimal addition level of EGCG was evaluated using quadratic regression. Pearson's correlation analysis was performed on the data that exhibited a linear relationship and normal distribution. Data visualization was performed using a mapping website (https://www.chiplot.online).

## 3. Results

### 3.1. Growth Performance

**Figure 1:**
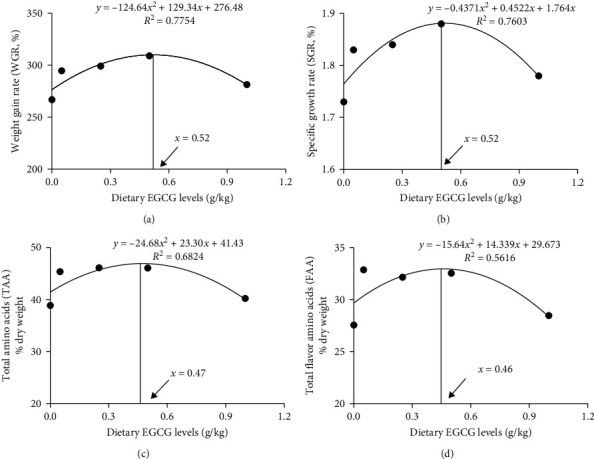
Quadratic regression analysis of percentage, WGR (a), SGR (b), TAA (c), and FAA (d) fed with graded dietary EGCG (0, 0.05, 0.25, 0.5, or 1 g/kg). Data are represented as means.

The survival rate of the carp was 100% following the 60-day feeding trial (Table 1). Compared to the CN group, the final body weight (FBW) and specific growth rate (SGR) exhibited a quadratic increase (*P* < 0.05), whereas the feed conversion ratio (FCR) exhibited a quadratic decrease (*P* < 0.05) with increasing EGCG from 0.05 to 0.5 g/kg. Additionally, the weight gain rate (WGR) and condition factor (CF) showed a quadratic increase (*P* < 0.01) with dietary EGCG levels supply ranging from 0.25 to 0.5 g/kg. However, neither the hepatosomatic index (HSI) nor the viscerosomatic index (VSI) varied significantly among diets. According to the quadratic regression analysis of WGR (Figure 1(a)) and SGR (Figure 1(b)), the optimal EGCG supplementation levels were 0.52 g/kg.

### 3.2. Antioxidant mRNA Levels and Enzyme Activities in the Muscle

**Figure 2:**
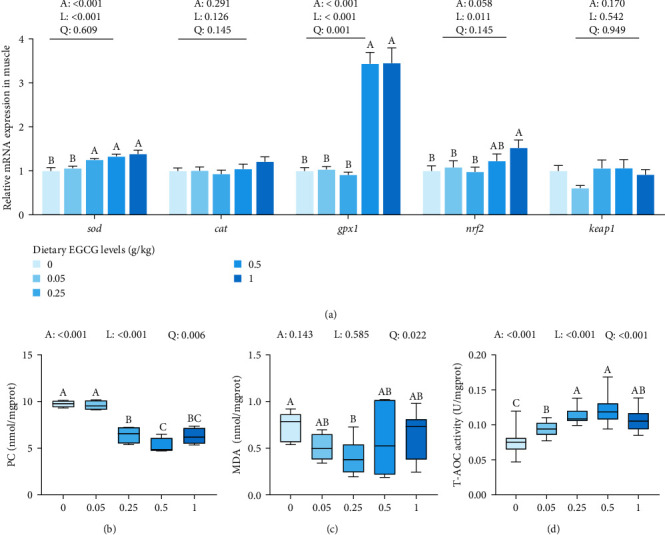
Effects of dietary EGCG on antioxidant parameters in the muscle of common carp (*C. carpio* L.) (*n* = 9). (a) The mRNA relative expression levels of antioxidant response-related genes. (b) proteincarbonyl (PC) contents. (c) malondialdehyde (MDA) contents. (d) total antioxidant capacity (T-AOC). Data are means ± SEM. One-way ANOVA followed by Duncan's test was used to analyze the discrepancy among all test groups. Different symbols denote significant differences (*P* < 0.05). The *P*-values indicate a significantly linear or quadratic dose–response relationship. Liner trend and quadratic trend were analyzed by polynomial contrasts (A, ANOVA; L, linear; Q, quadratic). Different column colors represent dietary EGCG supplemented at 0, 0.05, 0.25, 0.5, or 1 g/kg.

Figure 2(a) shows the antioxidant gene expression levels in the muscle. Superoxide dismutase (*sod*) gene expression levels showed a significant (*P* < 0.01) linear up-regulation as a dietary EGCG was increased from 0.25 to 1 g/kg. Similarly, glutathione peroxidase (*gpx*) mRNA level increased linearly with 0.5 and 1 g/kg EGCG supplementation (*P* < 0.01). Additionally, nuclear factor erythroid-2-related factor 2 (*nrf2*) mRNA levels linearly increased in the 1 g/kg EGCG supplementation group (*P* < 0.05). However, dietary EGCG supplementation did not significantly alter the mRNA expression levels of catalase (*cat*) or the kelch-like ECH-associated protein 1 (*keap1*).

As shown in Figure 2(b)–2(d), MDA content significantly (*P* < 0.05) decreased quadratically with dietary EGCG at 0.25 and 0.5 g/kg levels compared with the CN group, while a significant (*P* < 0.01) linear and quadratic decrease was observed in the PC content of muscle as dietary EGCG increased from 0.25 to 1 g/kg. Conversely, the total antioxidant capacity (T-AOC) of muscle exhibited a significant (*P* < 0.01) linear and quadratic increase with increasing dietary EGCG supplementation.

### 3.3. Fillet Composition and Physicochemical Indicators

According to the results shown in Table 2, the addition of EGCG did not significantly change the muscle moisture or crude ash content of common carp. However, compared to the CN group, the crude protein content significantly (*P* < 0.05) increased with dietary 0.05 g/kg EGCG supplementation, while the crude lipid content significantly (*P* < 0.05) decreased with 1 g/kg EGCG supplementation. In addition, the muscle cooking followed a significant (*P* < 0.01) quadratic decline in the groups supplemented with 0.05 and 0.5 g/kg of EGCG. Moreover, a significant (*P* < 0.05) quadratic decrease was observed in flesh drip loss with increasing EGCG supplementation from 0.05 to 0.5 g/kg. In turn, muscle pH increased linearly (*P* < 0.05) as the EGCG supplement increased from 0.25 to 1 g/kg. Conversely, lactate content exhibited a linearly significant (*P* < 0.01) decrease with 0.25 and 1 g/kg EGCG supplementation. Finally, the hydroxyproline content significantly (*P* < 0.05) decreased with the addition of 1 g/kg EGCG.

### 3.4. Muscle Histomorphology and Texture

**Figure 3:**
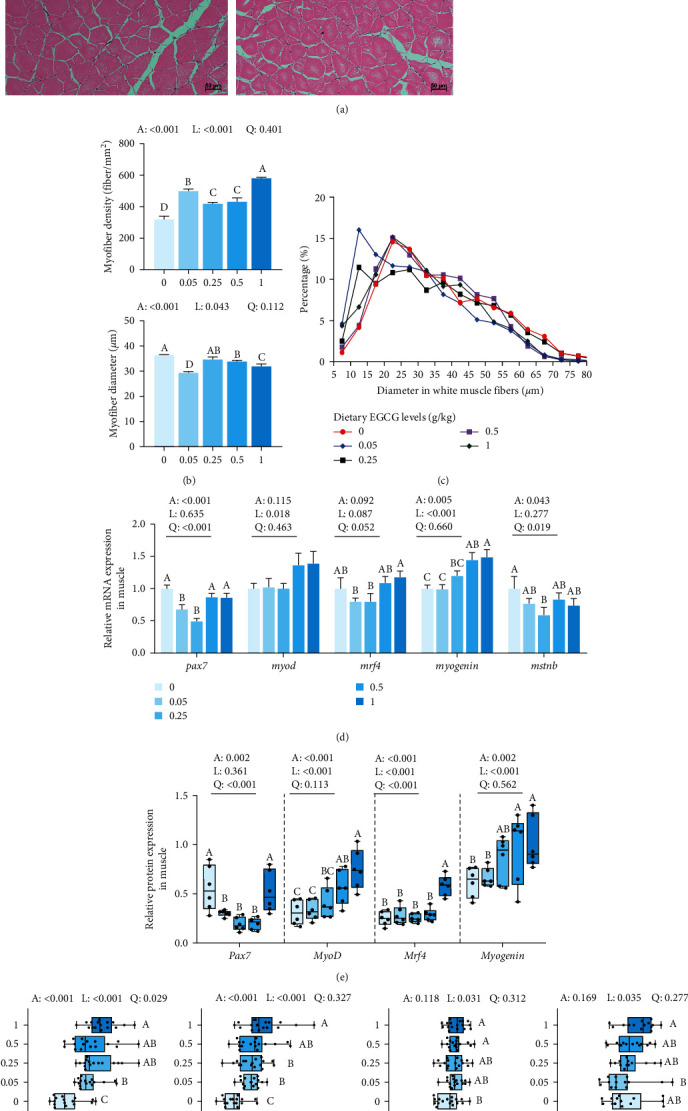
Effects of dietary EGCG on myofiber growth and the texture of raw fillet in common carp (*C. carpio* L.). (a) Transversal section of a dorsal white muscle fiber; (b) myofiber density and diameter (*n* = 3); (c) diameter distribution of the muscle myofiber (*n* = 3). Data are represented as mean; (d) muscle growth-related gene expressions (*n* = 9); (e) muscle fluorescence intensity (*n* = 6); (f) texture of raw fillet (*n* = 9). The data are represented as the mean ± SEM. One-way ANOVA followed by Duncan's test was used to analyze the discrepancy among all test groups. Different symbols denote significant differences (*P* < 0.05). *P*-values indicate a significantly linear or quadratic dose–response relationship. Liner trend and quadratic trend were analyzed by polynomial contrasts (A, ANOVA; L, linear; Q, quadratic). Different column or line colors represent dietary EGCG supplemented at 0, 0.05, 0.25, 0.5, or 1 g/kg.

The results in Figure 3(a)–3(c) show that myofiber density significantly (*P* < 0.01) increased with EGCG supplementation. The myofiber diameter showed a significant (*P* < 0.05) linear decrease with increasing 0.05, 0.5, and 1 g/kg EGCG supplementation.

As for fillet texture (Figure 3(f)), muscle hardness and chewiness were significantly (*P* < 0.01), enhanced by EGCG supplementation in a linear and quadratic pattern, respectively. Additionally, springiness was significantly (*P* < 0.05) enhanced with 0.5 and 1 g/kg EGCG supplementation compared with the CN group. However, shear force did not change remarkably with EGCG addition.

### 3.5. Myofiber Growth

The mRNA expression levels involved in myofiber growth in common carp are shown in Figure 3(d). Compared to the CN group, the mRNA level of paired box 7 (*pax7*) was significantly (*P* < 0.05) downregulated by 0.05 and 0.25 g/kg EGCG supplementation. Meanwhile, the *myogenin* mRNA was significantly (*P* < 0.01) upregulated with 0.5 and 1 g/kg EGCG supplementation in a linear and quadratic fashion, respectively. Conversely, compared to the CN group, dietary 0.25 g/kg EGCG supplementation significantly (*P* < 0.05) downregulated the mRNA level of myostatin b (*mstnb*).

Figure 3(e) and *Supplementary 3* show the Pax7, myogenic differentiation antigen (MyoD), myogenic regulatory factor 4 (Mrf4), and myogenin protein levels, as well as the immunofluorescent diagrams of white muscle. Pax7 protein expression level showed significant (*P* < 0.01) quadratic downregulation with EGCG supplementation, ranging from 0.05 to 0.5 g/kg. Futhermore, MyoD and Myogenin protein expression levels showed significant (*P* < 0.01) linear and quadratic increases with 0.5 and 1 g/kg EGCG supplementation, respectively. Similarly, Mrf4 protein expression level significantly (*P* < 0.01) increased linearly and quadratically in the 1 g/kg treatment group.

### 3.6. Amino Acid Metabolism in Carp Muscle

**Figure 4:**
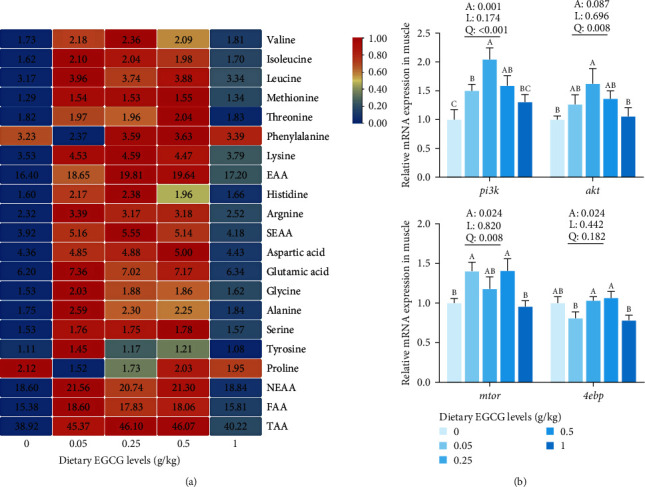
Effect of dietary EGCG on amino acids (heatmap visualization) and mRNA relative expression levels of protein synthesis-related genes in muscle of common carp (*C. carpio* L.). (a) Heatmap visualization of amino acids. Each row of the heatmap was labeled with amino acid names, and colors refer to the relative levels of these amino acids from high (red) to low (blue). Data are represented as means (*n* = 3). Detailed data are available in *Supplementary 4* (b) mRNA relative expression levels of protein synthesis-related genes. Data are means ± SEM (*n* = 9). One-way ANOVA followed by Duncan's test was used to analyze the discrepancy among all the groups. Different symbols denote significant differences (*P* < 0.05). The *P*-values indicate a significantly linear or quadratic dose–response relationship. Liner trend and quadratic trend were analyzed by polynomial contrasts (A, ANOVA; L, linear; Q, quadratic). Different column colors represent dietary EGCG supplemented at 0, 0.05, 0.25, 0.5, or 1 g/kg.

Figure 4(a) and *Supplementary 4* summarize the amino acid contents in carp muscle. EGCG supplementation levels from 0.05 to 0.5 g/kg in the diet showed highly significant (*P* < 0.01) quadratic effects on Val, Ile, Leu, Met, Thr, Lys, His, Arg, Asp, Glu, Gly, Ala, Ser, and Tyr contents. In turn, the contents of non-essential amino acids (NEAA), essential amino acids (EAA), semi-essential amino acids (SEAA), total flavor amino acids (FAA), and total amino acids (TAA) were significantly (*P* < 0.01) enhanced in a quadratic manner with EGCG levels from 0.05 to 0.5 g/kg. In addition, Val content significantly (*P* < 0.05) increased quadratically with 0.25 g/kg EGCG supplementation. Conversely, Pro content significantly (*P* < 0.05) decreased in the 0.05 and 0.25 g/kg EGCG treatment groups. Compared with the CN group, Phe content significantly (*P* < 0.05) decreased in the 0.05 g/kg EGCG group and increased in the 0.25 and 0.5 g/kg EGCG groups. According to the flavor amino acid (FAA) contents, an appropriate level of EGCG for common carp is predicted to be 0.46 g/kg, as shown in Figure 1(d).

Figure 4(b) shows that the mRNA expression levels of phosphatidylinositol 3 kinase (*pi3k*) and protein kinase B (*akt*) were significantly upregulated quadratically in the 0.25 g/kg EGCG supplementation group. In turn, the rapamycin (*mtor*) gene expression level was quadratically (*P* < 0.05) upregulated with 0.05 and 0.5 g/kg EGCG supplementation. However, the gene expression level of the eIF4E-binding protein (*4ebp*) showed no significant change with EGCG addition compared to the CN group.

### 3.7. Fatty Acid Deposition in the Carp Muscle

**Figure 5:**
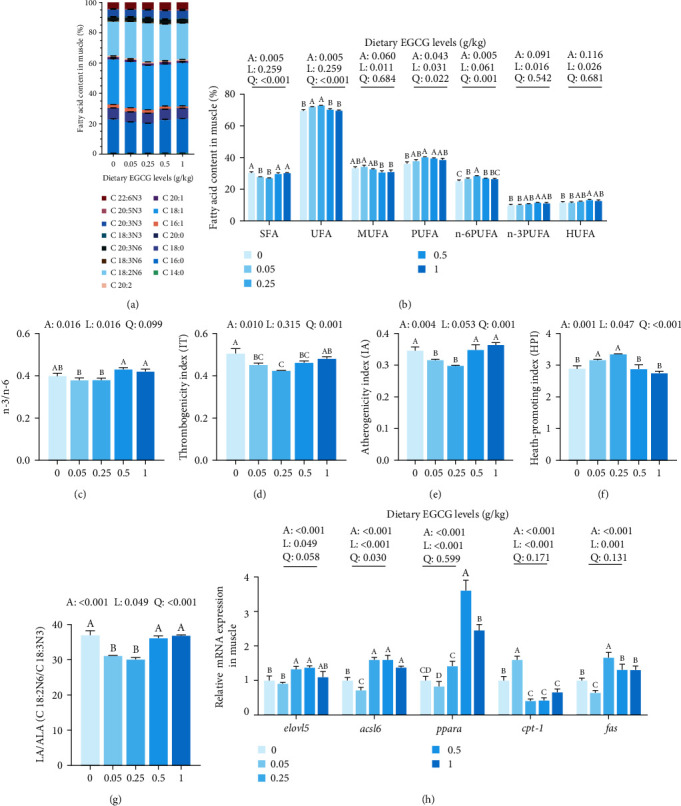
Effects of dietary EGCG on the muscle fatty acids of common carp (*C. carpio* L.). (a) Percentage of muscle amino acids in different treatment groups. Bars indicate the percentage value of fatty acids; (b) percentage of various types of fatty acids; (c) ratio of n-3 and n-6; (d) index of thrombogenicity (IT); (e) index of atherogenicity (IA); (f) index of health-promoting (HPI); (g) ratio of LA (C 18 : 2N6) and ALA (C18 : 3N3). Data are represented as mean ± SEM (*n* = 3); (h) the mRNA relative expression levels of fatty acid metabolism and lipid metabolism related genes (*n* = 9). One-way ANOVA followed by Duncan's test was used to analyze the discrepancy among all test groups. Different symbols denote significant differences (*P* < 0.05). *P*-values indicate a significantly linear or quadratic dose–response relationship. Liner trend and quadratic trend were analyzed by polynomial contrasts (A, ANOVA; L, linear; Q, quadratic). Different column colors represent dietary EGCG supplemented at 0, 0.05, 0.25, 0.5, or 1 g/kg.

Figure 5 and *Supplementary 5* show the composition and percentage of fatty acids in the muscle. Overall, linear and quadratic increases in unsaturated fatty acids (UFA) in the muscle were observed in the EGCG group. Conversely, dietary EGCG levels of 0.05 and 0.25 g/kg caused a quadratic decline in saturated fatty acids (SFA). Furthermore, the percentages of polyunsaturated fatty acids (PUFA) were significantly (*P* < 0.05) increased linearly and quadratically with 0.25 and 0.5 g/kg EGCG supplementation, respectively. Further, n-6 PUFA showed a highly significant (*P* < 0.01) increase with 0.05 and 0.5 g/kg EGCG supplementation.  Figure 5(b) and Table S4 show that the levels of C 22 : 6n3 (DHA), n-3 polyunsaturated fatty acids (n-3 PUFA), and high unsaturated fatty acids (HUFA) significantly (*P* < 0.05) increased with 0.5 g/kg EGCG supplementation compared with CN group. Meanwhile, the highest ratio of n-3 PUFA to n-6 PUFA was observed in the 0.5 g/kg EGCG treatment groups; however, there was no significant change compared with the CN group (Figure 5(c)).

Further analysis revealed a significant (*P* < 0.05) quadratic decrease in the atherogenicity index (IA) (Figure 5(e)) and LA/ALA ratio with EGCG supplementation (Figure 5(g)). The thrombogenicity index (IT) also showed a significant (*P* < 0.01) quadratic decrease with increasing EGCG supplementation from 0.05 to 0.5 g/kg (Figure 5(d)). Conversely, the health-promoting index (HPI) exhibited a significant (*P* < 0.01) quadratic increase with 0.05 and 0.25 g/kg EGCG supplementation (Figure 5(f)).

As shown in Figure 5(h), fatty acyl elongase 5 (*elovl5*) mRNA level was significantly (*P* < 0.01) and linearly enhanced with 0.25 and 0.5 g/kg EGCG supplementation. However, compared to the CN group, the long-chain fatty acyl-CoA synthetase 6 (*acsl6*) mRNA levels significantly (*P* < 0.05) decreased in the 0.05 g/kg EGCG group and significantly (*P* < 0.05) increased in the 0.25, 0.5, and 1 g/kg EGCG groups. Furthermore, the peroxisome proliferator-actiated receptor *α (pparα*) mRNA level exhibited a significant (*P* < 0.01) linear increase with 0.5 and 1 g/kg EGCG supplementation. In turn, the carnitine palmitoyl transferase 1 (*cpt-1*) expression level was upregulated in the 0.05 g/kg group but downregulated with 0.25, 0.5, and 1 g/kg EGCG supplementation significantly (*P* < 0.05). Finally, the mRNA level of fatty acid synthetase (*fas*) exhibited significant (*P* < 0.05) downregulation with 0.05 g/kg EGCG supplementation and upregulation with 0.25 g/kg EGCG supplementation.

### 3.8. Correlation Analysis

**Figure 6:**
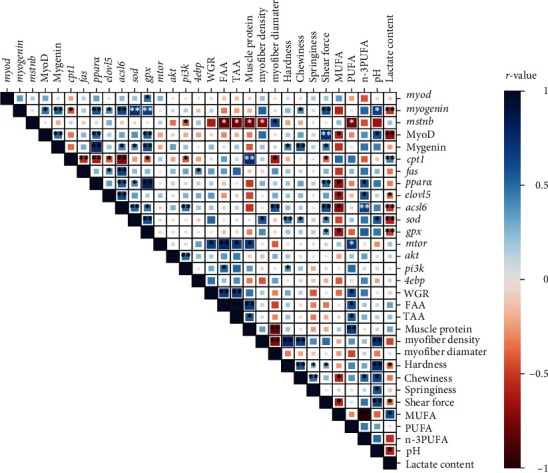
Correlation analysis between important parameters related to muscle quality of common carp ( ^*∗∗*^indicates *P* < 0.01;  ^*∗*^indicates *P* < 0.05, and color intensity indicates correlation value). Dietary EGCG was supplemented at 0, 0.05, 0.25, 0.5, or 1 g/kg.

As shown in Figure 6, the WGR of common carp was positively correlated with *mtor* mRNA expression levels, TAA, FAA, and PUFA (*r* = + 0.666, *P*=0.018; *r* = + 0.692, *P*=0.013; *r* = + 0.779, *P*=0.003; *r* = + 0.703, *P*=0.011, respectively). In turn, muscle protein content positively correlated with the expression of *mtor*, *cpt1*, TAA, and FAA (*r* = + 0.663, *P*=0.019; *r* = + 0.780, *P*=0.005; *r* = + 0.546, *P*=0.035; *r* = + 0.704, *P*=0.003, respectively). However, muscle protein was significantly and negatively correlated with *mstnb* gene level (*r* = − 0.735, *P*=0.024). Meanwhile, n-3PUFA levels positively correlated with the gene expression levels of *ppara*, *elovl5*, and *acsl6* (*r* = + 0.569, *P*=0.042; *r* = + 0.575, *P*=0.04; *r* = + 0.668, *P*=0.009, respectively). Further, muscle chewiness was positively correlated with myofiber density, protein expression level of Myogenin, and gene expression level of *sod* (*r* = + 0.663, *P*=0.007; *r* = + 0.573, *P*=0.001; *r* = + 0.400, *P*=0.021, respectively). Lastly, there was a significant negative correlation between muscle pH and lactic acid content (*r* = − 0.557, *P*=0.031).

## 4. Discussion

During periods of intensive feeding, transportation, and harvesting, fish are susceptible to oxidative stress, which can result in a loss of muscle quality. This study revealed that EGCG enhanced the muscle quality of common carp, particularly in terms of muscle growth, flavor amino acid contents, fatty acid deposition, physicochemical properties, and antioxidant regulation capacity.

### 4.1. EGCG Supplementation Promoted the Growth of Common Carp

This study demonstrated that an optimal level (0.52 g/kg) of EGCG had beneficial effects on weight gain in common carp. Previous studies have reported similar findings in grass carp [7], rainbow trout (*Oncorhynchus mykiss*) [18], and GIFT tilapia [8]. Correlation analysis showed that WGR was significantly and positively correlated with *mtor* gene expression. Therefore, growth promotion by EGCG in common carp may be related to the activation of the mTOR pathway. Muscle growth is a critical factor in determining fish growth performance. This study focused on the effects of dietary EGCG supplementation on muscle growth, muscle nutritional value, and health indices in common carp.

### 4.2. EGCG Supplementation Enhanced the Antioxidant Ability in Common Carp Muscle

Fish muscle is a rich source of protein and lipids (particularly unsaturated ones). Proteins and lipids are susceptible to oxidation by ROS. In particular, MDA serves as an important marker of cellular oxidative damage and the extent of hepatopancreatic damage. Additionally, PC is the primary biomarker of protein oxidation in animal muscles. The results of this study revealed that muscle levels of MDA and PC significantly decreased with intermediate or high doses of EGCG in the growth medium. Similar results have been observed in the large yellow croaker (*Larimichthys crocea*) [12], juvenile Japanese seabass (*Lateolabrax japonicus*) [33], and the Chinese Rice Field Eel (*Monopterus albus*) [16]. These results were attributed to the potent antioxidant effects of EGCG. Previous studies have suggested that EGCG has a stronger antioxidant capacity than vitamin E [18] or flavonoids [34], and the activity of antioxidant enzymes is reportedly influenced by the expression of specific genes [35]. Specifically, previous studies showed that 80 mg/kg of tea polyphenols up-regulate antioxidant-related genes by activating the Nrf2/keap1 pathway [14]. Consistently, this study showed that dietary EGCG supplementation enhanced the expression of *sod*, *gpx*, and *nrf2* in the muscle. Antioxidants reportedly improve the antioxidant ability of muscles in vitro, thereby inhibiting the development of undesirable sensory properties [36, 37]. Therefore, our subsequent step was to explore whether adding EGCG to the diet could enhance the physical and chemical properties of muscles.

### 4.3. EGCG Supplementation Improved the Physical and Chemical Indicators of Common Carp

Fish flesh quality is influenced by changes in metabolism and physical and chemical properties. Drip loss, cooking loss, pH, and lactate content are vital physicochemical indicators for evaluating the sensory quality of fish flesh [10]. Muscle pH was highest in muscles with a dietary EGCG level of 0.25 g/kg, presumably owing to the resulting reduction in lactate content. Correlation analysis showed that pH was significantly and negatively associated with muscle lactate levels. Similarly, Most et al. [38] reported that 3-day supplementation with EGCG reduced lactate levels in human skeletal muscle. Specifically, EGCG stimulates aerobic metabolism and inhibits anaerobic metabolism in muscles. Similarly, other plant extracts, such as quercetin [39], resveratrol [40], *E. ulmoides* extracts [41], and grape seed proanthocyanidin extract [42], reportedly decrease the glycolytic potential of muscles and enhance aerobic metabolism in fish, pigs, and broilers, resulting in reduced lactate levels.

Low drip and cooking losses are important factors that reflect high flesh quality, as they indicate water-holding capacity and juiciness. This study demonstrated that dietary EGCG reduced both muscle cooking and drip loss. Similarly, in a previous study, tea polyphenol was also reported to reduce muscle cooking loss in grass carp [7]. Indeed, EGCG reportedly has a cross-linking effect on common carp myofibrillar proteins, thereby leading to a decrease in drip and cooking losses [43].

The sensory quality of fish meat is determined by its hardness, chewiness, springiness, and shear force. This study indicated that the EGCG supplementation increased fillet hardness and chewiness, both of which are associated with muscle lipid contents, collagen contents, and fiber characteristics. In this study, the lipid and collagen contents in the muscle were significantly decreased with the addition of EGCG at high concentration, while myofiber density significantly increased. Enhanced muscle hardness and chewiness may be related to muscle lipid content and myofiber density. Indeed, previous reports have suggested that myofiber density is directly proportional to muscle hardness [21, 44–46]. Similarly, we found that EGCG caused a significant increase in myofiber density and a concomitant decrease in myofiber diameter. Correlation analysis revealed that muscle fiber density was significantly and positively connected with hardness and chewiness. Therefore, this study focused on the effects of EGCG on muscle fiber growth.

### 4.4. EGCG Supplementation Improved Common Carp Muscle-Fiber Development

Muscle fiber characteristics are strongly associated with skeletal muscle growth. Unlike the case in mammals, in certain teleost species, muscle fiber hyperplasticity and hypertrophy occur during growth. Skeletal muscle growth is a process whereby myoblasts proliferate and differentiate to form myotubes and eventually myofibers. Thus, during this period, it is regulated by myogenic regulatory factors (MRFs), which include the positive regulators Myf5, Myod, and Mrf4, Myogenin, and the negative regulator MSTN. In particular, MyoD is widely recognized as the primary regulator of cell proliferation and differentiation during the initial phases of skeletal muscle development [47]. In turn, myogenin and MRF4, the principal regulators of skeletal muscle structural genes, play crucial roles in the terminal differentiation of myofiber [48]. As for Pax7, it is expressed in satellite cells of skeletal muscles attached to resting muscle fibers; furthermore, studies have shown that pax7 is highly expressed in undifferentiated muscle cells of trout, whereas its expression is reduced in differentiated myogenic cells [49]. Interestingly, in this study, supplementation with a low dose of EGCG resulted in a significant downregulation of pax7. However, the protein expression levels of Myod, Mrf4, and Myogenin were linearly enhanced in the high-dose EGCG treatment group. This finding may be due to EGCG actively prompting more cells to enter the differentiation stage. Conversely, MSTN negatively regulates muscle development. In mammals, EGCG inhibits MSTN expression by regulating the expression of miRNA-485-5p, thereby alleviating age-induced skeletal muscle atrophy [50]. Here, we found that appropriate doses of EGCG inhibited the expression of *mstnb*, which, in turn, contributed to muscle growth. This finding agreed closely with some studies that have reported that *salvia miltiorrhiza* leaves promote muscle growth and protein synthesis in common carp by inhibiting MSTN expression [51]. Therefore, EGCG seemingly promoted muscle growth by promoting muscle cell differentiation and inhibiting *mstnb* expression. However, the molecular mechanisms at play at the cellular level warrant further research. As inhibition of MSTN expression promotes muscle protein synthesis, we explored the effect of EGCG on muscle nutrient deposition in carp.

### 4.5. EGCG Supplementation Improved Muscle Nutrient Deposition in Common Carp

Fish is a high-protein food, and specifically, fish protein level directly represents the nutritional value of fish muscle. In particular, the PI3K/AKT/mTOR signaling pathway plays a crucial role in the regulation of muscle protein synthesis and muscle growth [52]. Coincidentally, EGCG promotes muscle protein synthesis in common carp, which may be related to the PI3K/AKT/mTOR signaling pathway. Correlation analysis also showed that *mtor* expression levels were significantly and positively correlated with crude protein and TAA contents in the muscle. Furthermore, amino acids are not only the main elements of protein synthesis, but they affect meat flavor as well. Thus, to determine the effects of EGCG on flesh flavor, we examined the amino acid content of common carp muscle. Lysine and methionine are essential amino acids known to play important roles in physical growth. Furthermore, glutamic, aspartic, glycine, serine, and alanine are considered flavor amino acids in muscle. In particular, glutamic and aspartic acids contribute directly to the umami taste, whereas glycine, serine, and alanine are considered sweet amino acids [4]. Interestingly, this study indicated that optimal EGCG supplementation improved fish flesh taste quality. Previous studies reported that some plant extracts, such as rutin [53], catechins [54], and grape seed proanthocyanidin extract [9], all increase FAA content in the muscles of grass carp and Nile tilapia. Furthermore, it is well known that amino acid metabolism is closely related to glucose metabolism and that insulin has a profound influence on the metabolism of amino acids and proteins. Tea polyphenols reportedly regulate glucose metabolism and reduce blood glucose levels in grass carp [55]. In general, a decrease in blood glucose levels is accompanied by an increase in plasma insulin levels. One possible explanation for the observed enhanced amino acid content in the muscle is that increasing insulin promotes the transfer of amino acids from the blood into the muscle [56, 57]. In animals, amino acid uptake and nitrogen metabolism are the two main metabolic pathways involved in controlling muscle amino acid metabolism and protein accumulation. Studies have reported that polyphenols, such as chlorogenic acid [58] and apple polyphenols [59], induce an increase in the concentration of free amino acids in the blood and muscle of pigs and reduce nitrogen release, thereby enhancing the changes in amino acid transporters and promoting protein synthesis. Therefore, we speculated that the increase in muscle amino acid content might be related to the regulation of glucose and nitrogen metabolism by EGCG. However, the underlying mechanisms warrant further research.

In fish, muscle fatty-acid composition and content also reflect the flavor and nutritional value of the flesh [4]. Fish are hailed as health foods by consumers because they contain abundant polyunsaturated fatty acids (PUFAs). There are many indices for evaluating muscle health, all of which are based on muscle fatty acid composition and content. First, the IA value characterizes the ratio of SFA to UFA. Lower SFA levels and higher UFA levels in foods are effective in preventing chronic diseases [60]. In this study, dietary EGCG supplementation with 0.05 and 0.25 g/kg resulted in a decrease in the IA value. In fish, a higher proportion of n-3/n-6 PUFA and a higher HUFA content in meat are generally considered beneficial for health. This study showed that supplementation with 0.5 g/kg EGCG resulted in an increase in the rate of HUFA and the value of n-3/n-6. Second, the IT value represents the ratio between prothrombogenic and antithrombogenic fatty acids in the muscle. The lower the IT value of the muscle, the more beneficial it is for cardiovascular health. The findings of this study demonstrated that dietary EGCG levels ranging from 0.05 to 0.5 g/kg resulted in a decrease in IT value in flesh. ALA (C18 : 3n-3) is a crucial fatty acid in the human body that is responsible for synthesizing EPA and DHA through multiple saturation and extension reactions [61]. However, LA (C18 : 2n-6) competes with ALA for desaturase and elongation enzymes, resulting in the inhibition of the synthesis of long-chain unsaturated fatty acids [3]. This study revealed that adding an appropriate amount of EGCG to the diet reduced the LA/ALA ratio in fish flesh. Ultimately, the health value of fish flesh can be represented by the HPI. This study suggested that optimal EGCG supplementation enhanced fish flesh health. In mammals, previous studies have reported that polyphenols improve the ratio of PUFA to SFA in muscles [59]. A possible explanation for these results is that EGCG promotes fatty acid *β*-oxidation and lipid catabolism. Therefore, this study aimed to evaluate the expression levels of genes involved in muscle fatty acid metabolism and deposition. Specifically, Acsl6 is an enzyme that plays an essential role in DHA synthesis and muscle deposition [62]. Elovl5 is one of several rate-limiting enzymes involved in the synthesis of LC-PUFA [63]. In addition, PPAR*α* activates fatty acid *β* oxidation, thereby regulating fatty acid metabolism. In this study, EGCG supplementation significantly upregulated *elovl5*, *acsl6*, and *pparα* mRNA levels, which may have led to an increase in muscle n-3 PUFA proportion in common carp. A similar phenomenon has been observed in large yellow croaker [12] and grass carp [54]. These results indicate that an appropriate dietary EGCG level may positively regulate the muscle fatty acid metabolism in common carp.

## 5. Conclusions

The results summarized herein indicate that EGCG supplementation in the diet improved the growth of juvenile common carp. Specifically, dietary EGCG supplementation had the following beneficial effects on the muscles of common carp: (1) it enhanced muscle antioxidant capacity and reduced drip loss, cooking loss, lactate content, MDA content, and PC content; (2) it increased muscle nutritional value, including higher contents of muscle protein, FAA, TAA, and n-3 PUFA; (3) it increased myofiber density, muscle chewiness, and the expression levels of Myod, Myogenin, and *mrf4*. However, a high dose of EGCG supplementation in diets contributed to a decrease in muscle crude lipid and hydroxyproline contents. Therefore, the optimal EGCG supplementation level for maximum growth and muscle animo acid deposition of common carp is in the range of 0.46–0.52 g/kg (Figure 1). These results provide a solid theoretical foundation for using EGCG in common carp culture to produce healthy and high-nutritional quality fish flesh.

## Figures and Tables

**Table 1:** Effects of dietary EGCG on growth indexes of common carp (*C. carpio* L.).

Parameters	Dietary EGCG levels (g/kg)					*P*-value		
	0	0.05	0.25	0.5	1	A	L	Q
IBW (g)	17.38 ± 0.06	17.46 ± 0.19	17.25 ± 0.38	17.18 ± 0.09	17.38 ± 0.19	0.879	0.684	0.640
FBW (g)^1^	63.76 ± 0.73^c^	68.91 ± 0.49^ab^	68.77 ± 0.64^ab^	70.28 ± 1.22^a^	66.27 ± 1.34^bc^	0.005	0.058	0.001
WGR (%)^2^	266.88 ± 5.54^b^	294.71 ± 7.09^ab^	299.14 ± 9.99^a^	309.12 ± 6.97^a^	281.49 ± 11.62^ab^	0.043	0.137	0.008
SGR (%/d)^3^	1.73 ± 0.02^b^	1.83 ± 0.02^a^	1.84 ± 0.03^a^	1.88 ± 0.02^a^	1.78 ± 0.04^ab^	0.041	0.135	0.008
FCR^4^	1.40 ± 0.03^a^	1.25 ± 0.02^bc^	1.25 ± 0.02^bc^	1.21 ± 0.03^c^	1.32 ± 0.04^ab^	0.007	0.063	0.001
SR (%)^5^	100 ± 0.00	100 ± 0.00	100 ± 0.00	100 ± 0.00	100 ± 0.00	—	—	—
CF (g/cm^3^)^6^	2.41 ± 0.06^c^	2.41 ± 0.05^c^	2.66 ± 0.05^a^	2.63 ± 0.05^ab^	2.48 ± 0.07^bc^	0.006	0.057	0.010
HSI (%)^7^	1.73 ± 0.14	1.37 ± 0.17	1.63 ± 0.11	1.65 ± 0.17	1.62 ± 0.12	0.461	0.897	0.414
VSI (%)^8^	6.75 ± 0.27	6.51 ± 0.28	6.44 ± 0.26	6.19 ± 0.22	6.35 ± 0.35	0.705	0.213	0.559

^1^Final body weight (FBW, g) = final body weight/final number of fish; ^2^Weight gain rate (WGR, %) = 100 × (final body weight−initial body weight)/initial body weight; ^3^Specific growth rate (SGR, % day^−1^) = 100 × (Ln final individual weight−Ln initial individual weight)/number of feeding days; ^4^Feed conversion ratio (FCR) = feed consumed/weight gain; ^5^Survival rate (SR, %) = 100 × (final number of fish)/(initial number of fish); ^6^Condition factor (CF, g/cm ^3^) = 100 × body weight/body length^3^; ^7^Hepatosomatic index (HSI, %) = 100 × hepatosomatic wet weight/body wet weight; ^8^Viscerosomatic index (VSI, %) = 100 × (viscera weight, g)/(whole bodyweight, g); ^1–5^Values are means ± SEM (*n* = 3); ^6–8^Values are means ± SEM (*n* = 9). One-way ANOVA followed by Duncan's test was used to analyze the discrepancy among all the groups. Values in the same row with different superscripts represent statistically significant difference (*P* < 0.05). The *P*-values indicate a significantly linear or quadratic dose–response relationship (*P* < 0.05). Liner trend and quadratic trend were analyzed by polynomial contrasts (A, ANOVA; L, linear; Q, quadratic). EGCG was supplemented at 0, 0.05, 0.25, 0.5, or 1 g/kg.

**Table 2:** Effects of dietary EGCG on muscle composition and physicochemical properties of common carp muscle (*C. carpio* L.).

Parameters	Dietary EGCG levels (g/kg)					*P*-value		
	0	0.05	0.25	0.5	1	A	L	Q
Dry matter (%)^1^	20.63 ± 0.85	20.98 ± 0.10	20.58 ± 0.12	20.33 ± 0.14	20.62 ± 0.32	0.868	0.624	0.988
Crude protein (% DM)^2^	82.26 ± 0.28^c^	86.61 ± 0.47^a^	83.59 ± 0.51^bc^	84.18 ± 0.51^b^	83.38 ± 0.84^bc^	0.003	0.916	0.009
Crude lipid (% DM)^3^	4.75 ± 0.10^a^	4.51 ± 0.06^a^	4.56 ± 0.08^a^	4.59 ± 0.03^a^	3.94 ± 0.20^b^	<0.001	<0.001	0.022
Crude Ash (% DM)^4^	8.22 ± 0.84	7.71 ± 0.98	8.17 ± 0.59	7.70 ± 0.41	7.07 ± 0.18	0.748	0.300	0.651
Cooking loss (%)^5^	17.41 ± 0.58^a^	14.60 ± 0.67^b^	16.08 ± 0.49^ab^	14.72 ± 0.87^b^	17.32 ± 0.51^a^	0.007	0.970	0.002
Drip loss (%)^6^	21.60 ± 0.68^a^	17.50 ± 0.71^b^	18.65 ± 1.46^b^	18.42 ± 0.21^b^	21.34 ± 0.45^a^	0.016	0.870	0.002
pH^7^	6.65 ± 0.06^c^	6.83 ± 0.05^bc^	6.89 ± 0.05^ab^	6.85 ± 0.08^ab^	7.05 ± 0.02^a^	0.011	0.001	0.673
Lactate content (mmol/gprot)^8^	1.34 ± 0.02^a^	1.33 ± 0.04^a^	1.21 ± 0.04^b^	1.26 ± 0.03^ab^	1.11 ± 0.03^c^	<0.001	<0.001	0.365
Hydroxyproline (*μ*g/mg tissue)^9^	0.53 ± 0.02^a^	0.51 ± 0.02^ab^	0.55 ± 0.02^a^	0.49 ± 0.02^ab^	0.47 ± 0.01^b^	0.041	0.030	0.151

^1–4^Values are means ± SEM (*n* = 3). ^5–7^Values are means ± SEM (*n* = 6). ^8–9^Values are means ± SEM (*n* = 9). One-way ANOVA followed by Duncan's test was used to analyze the discrepancy among all the groups. Values in the same row with different superscripts represent statistically significant difference (*P* < 0.05). The *P*-values indicate a significantly linear or quadratic dose–response relationship (*P* < 0.05). Liner trend and quadratic trend were analyzed by polynomial contrasts (A, ANOVA; L, linear; Q, quadratic). EGCG was supplemented at 0, 0.05, 0.25, 0.5, or 1 g/kg.

## Data Availability

The datasets are included in this article and available from the corresponding author on reasonable request.

## Supplementary Materials

Supplementary 1 Composition of ingredients and nutrient content of experimental diets in feeding trial (g/kg, dry matter).

Supplementary 2 Real-time PCR primer sequences used.

Supplementary 3 Immunofluorescence diagrams of Pax7, MyoD, Mrf4, and Myogenin in the white muscle of common carp.

Supplementary 4 Effect of dietary EGCG on the amino acid composition of white muscle of common carp (*C. carpio* L.) (g/kg, dry matter).

Supplementary 5 Effect of dietary EGCG on the fatty acid content in the muscle of common carp (*C. carpio* L.) (% total fatty acid of dry matter).
