# Supplementary material for: (-)-Epigallocatechin-3-O-Gallate Regulates Muscle Growth, Antioxidant Status, and Nutritional Composition of Juvenile Common Carp (Cyprinus carpio L.)
Source: Aquac Nutr. 2024 Mar 20;2024:7134404. doi: 10.1155/2024/7134404 (PMC10977338; doi:10.1155/2024/7134404)
Supplement: Supplementary 1 — Composition of ingredients and nutrient content of experimental diets in feeding trial (g/kg, dry matter). [file 7134404.f1.docx]

**Table S1. Ingredient composition and nutrient content of experimental diets in feeding trial (g/kg, dry matter).**

| **Ingredients (g/kg)** | Dietary EGCG levels (g/kg) | | | | |
| --- | --- | --- | --- | --- | --- |
|  | 0 | 0.05 | 0.25 | 0.5 | 1 |
| Soybean meal ^1^ | 370 | 370 | 370 | 370 | 370 |
| Cottonseed meal ^2^ | 90 | 90 | 90 | 90 | 90 |
| Rapeseed meal ^3^ | 230 | 230 | 230 | 230 | 230 |
| Fish meal ^4^ | 100 | 100 | 100 | 100 | 100 |
| Wheat meal ^5^ | 45 | 45 | 45 | 45 | 45 |
| Soybean oil ^6^ | 75 | 75 | 75 | 75 | 75 |
| Ca (H_2_PO_4_)_2_ ^7^ | 25 | 25 | 25 | 25 | 25 |
| Vitamin premix ^8^ | 20 | 20 | 20 | 20 | 20 |
| Mineral premix ^9^ | 20 | 20 | 20 | 20 | 20 |
| Microcrystalline cellulose ^10^ | 25 | 24.5 | 24.75 | 24.5 | 24 |
| Epigallocatechin-3-O-gallate ^11^ | 0 | 0.05 | 0.25 | 0.5 | 1 |
| Epigallocatechin-3-O-gallate ^12^ | 0 | 0.049 | 0.240 | 0.494 | 0.949 |
| Total | 1000 | 1000 | 1000 | 1000 | 1000 |
| Proximate composition ^13^ | | | | | |
| Crude protein | 360.5 | 347.4 | 348.8 | 367.9 | 356.9 |
| Crude lipid | 98.5 | 99.1 | 96.6 | 103.4 | 99.8 |
| Crude ash | 132.7 | 144.5 | 146.5 | 124.5 | 144.8 |
| Moisture | 86.9 | 88.7 | 88.3 | 88.4 | 86.6 |

^1^ Provided by Henan Tongwei Co., Ltd., Xinxiang, China. 64 % crude protein.

^2^ Provided by Henan Hefeng Co., Ltd., Tangshan, China. 15.7 % crude protein.

^3^ Provided by Henan Hefeng Co., Ltd., Tangshan, China. 43.5 % crude protein.

^4^ Provided by Henan Tongwei Co., Ltd., Xinxiang, China. 44.2 % crude protein.

^5^ Provided by Henan Wudeli Co., Ltd., Xinxiang, China. 13.4 % crude protein.

^6^ Provided by Shandong Luhua Group Co., Ltd., China.

^7,8^ Provided by Henan Tongwei Co., Ltd., Xinxiang, China.

^9^ Provided by Henan Tongwei Co., Ltd., Xinxiang, China. Vitamin premix (g/kg premix): Retinyl acetate, 1.2121; cholecalciferol, 1.2000; all-rac-a-tocopherol, 20.0000; menadione, 9.0909; thiamine, 10.8696; Riboflavin, 7.5000; ascorbicacid, 30.0000; pyridoxinehydrochloride, 12.1212; cyanocobalamin, 2.0000; folicacid, 40.0000; biotin, 12.5000; nicotinicacid, 40.4040; D-Capantothenate, 16.1290; inositol, 204.0816; cellulose, 592.8915.

^10^ Provided by Henan Tongwei Co., Ltd., Xinxiang, China. Mineral premix (g/kg premix): FeC_6_H_5_O_7_, 11.43; ZnSO_4_⋅7H_2_O, 11.79; MnSO_4_⋅H_2_O (99 %), 2.49; CuSO_4_⋅5H_2_O (99 %), 1.06; MgSO_4_⋅7H_2_O (99 %), 27.31; KH_2_PO_4_, 233.2; NaH_2_PO_4_, 228.39; C_6_H_10_CaO_6_⋅5H_2_O (98 %), 34.09; CoCl_2_⋅6H_2_O (99 %), 1.36.

^11^ Provided by Nanjing Daosifu Biotech Co., Ltd., Nanjing, China; purity ≥ 98%.

^12^ The measured value of EGCG by the method of High-performance liquid chromatography (HPLC), The method of HPLC was based on SN/T 3848-2014 with some minor modifications.

^13^ Crude protein, lipid, and ash levels were measured value.
