# Supplementary material for: (-)-Epigallocatechin-3-O-Gallate Regulates Muscle Growth, Antioxidant Status, and Nutritional Composition of Juvenile Common Carp (Cyprinus carpio L.)
Source: Aquac Nutr. 2024 Mar 20;2024:7134404. doi: 10.1155/2024/7134404 (PMC10977338; doi:10.1155/2024/7134404)
Supplement: Supplementary 2 — Real-time PCR primer sequences used. [file 7134404.f2.docx]

**Table S2. Real-time PCR primer sequences used.**

| **Gene** | **Sequence (5′–3′)** | **Genbank ID** | **Efficiency**  **%** | **Size (bp)** |
| --- | --- | --- | --- | --- |
| *sod* F^1^ | GTCCGCACTTCAACCCTCAT | XM 019111527.2 | 96.4 | 169 |
| *sod* R | ATGGTCCTCCCAATGACCGA |  |  |  |
| *cat*F^2^ | TTCCTGTGGGACGCCTTGT | JF411604.1 | 95.1 | 170 |
| *cat*R | TCCGAGCCGATGCCTATGT |  |  |  |
| *gpx*F^3^ | AACCAGTTCGGACATCA | GQ376155.1 | 95.0 | 204 |
| *gpx*R | ATCACCCATCAAGGACA |  |  |  |
| *keap1*F^4^ | CAGTGGGCGAGAAGTGT | JX470752.1 | 91.4 | 112 |
| *keap1*R | TTTGATGGCTCCAGGTT |  |  |  |
| *nrf2*F^5^ | ACGACAAATGCCGAAGT | JX462955.1 | 93.4 | 115 |
| *nrf2*R | CTGCCTCATCTAGTGGAAA |  |  |  |
| *pax7F*^6^ | GCTCCATTAGTCGGGTTC | XM_042766854.1 | 94.4 | 166 |
| *pax7R* | GGCTCCGACTCCACATC |  |  |  |
| *MyodF*^7^ | CAACGACACGCCAAAT | XM_019068329.2 | 97.8 | 122 |
| *MyodR* | CTGACAGCACGGGACA |  |  |  |
| *Mrf4F*^8^ | TGTCTTATGTGGGCTTGT | XM_019092292.2 | 93.0 | 152 |
| *Mrf4R* | CTCTGGTTCGGATTGG |  |  |  |
| *myogeninF*^9^ | GAAGGCGGCGATAACTTC | XM_019096789.2 | 98.7 | 189 |
| *myogeninR* | CTGCTGCTCCTGGTGAG |  |  |  |
| *mstnbF*^10^ | AACTCCGACTCAAACAGG | XM_042764169.1 | 95.5 | 166 |
| *mstnbR* | ATGGTCTCAGTGGTGGC |  |  |  |
| *pi3kF*^11^ | GAAGATGACGACTGGAGAG | XM_019085737.2 | 104.1 | 400 |
| *pi3kR* | GCCTGTAGTGACTGATGAG |  |  |  |
| *aktF*^12^ | ACTGTTATTGAGCGCACCTT | XM_042774924.1 | 97.2 | 116 |
| *aktR* | TCCATTGGCTCCTCCTCTTC |  |  |  |
| *mtorF*^13^ | ATCATACGCATCCAGTCCATTG | FJ899680.1 | 102.1 | 190 |
| *mtorR* | GGTCATTAGCCAGTAGAGTGTTC |  |  |  |
| *4ebpF*^14^ | TCCTGGAGGCACTTTAT | XM_019083109.1 | 97.8 | 106 |
| *4ebpR* | TGGTGGGGTCTGGGCGAT |  |  |  |
| *elovl5F*^15^ | GAGGA TGGCTTCTACTG | MK893918.1 | 94.8 | 204 |
| *elovl5R* | ATCTGCCTTGATACACT |  |  |  |
| *acsl6F*^16^ | TTCCTTCCTGCCTCTTG | MF002109.1 | 98.5 | 248 |
| *acsl6R* | CCAGCCCTTTCCTCTTT |  |  |  |
| *pparαF*^17^ | GCGTGCTTTGGCTTTGTT | FJ849065.1 | 91.2 | 105 |
| *pparαR* | GGGAAAGAGCAGCACGAG |  |  |  |
| *cpt-1F*^18^ | CAGATGGAAAGTGTTGCTAATGAC | JQ361077.1 | 92.0 | 168 |
| *cpt-1R* | TGTGTAGAAGTTGCTGTTGACCA |  |  |  |
| *fasF*^19^ | GACAGGCCGCTATTGCTATT | GQ466045.1 | 95.5 | 110 |
| *fasR* | TGCCGTAAGCTGAGGAAATC |  |  |  |
| *18sF*^20^ | GAGACTCCGGCTTGCTAAAT | FJ710826.1 | 94.1 | 107 |
| *18sR* | CAGACCTGTTATTGCTCCATCT |  |  |  |

^1^ sod: superoxide dismutase;^2^ *cat*, catalase; ^3^ *gpx*, glutathione peroxidase; ^4^ *keap1*, kelch-like ECH-associated protein 1; ^5^ *nrf2*, nuclear factor erythroid-2-related factor 2; ^6^ *pax7,* paired box 7;^7^ *myod,* myogenic differentiation antigen; ^8^ *mrf4,* myogenic regulatory factor 4; ^9^ *Myogenin,* myogenin; ^10^ *mstnb,* myostatin b; ^11^*pi3k,* phosphoinositide 3-kinase; ^12^*akt,* protein kinase B; ^13^ *mtor,* target of rapamycin; ^14^ *4ebp,* eIF4E-binding protein; ^15^ *elovl5,* fatty acyl elongase 5; ^16^ *acsl6,* long-chain fatty acyl-CoA synthetase 6; ^17^ *pparα,* peroxisome proliferator activated receptor α; ^18^ *cpt-1,* carnitine palmitoyltransferase 1; ^19^ *fas,* fatty acid synthetase; ^20^*18s*,*18s* ribosomal RNA.
