# Supplementary material for: (-)-Epigallocatechin-3-O-Gallate Regulates Muscle Growth, Antioxidant Status, and Nutritional Composition of Juvenile Common Carp (Cyprinus carpio L.)
Source: Aquac Nutr. 2024 Mar 20;2024:7134404. doi: 10.1155/2024/7134404 (PMC10977338; doi:10.1155/2024/7134404)

**Fig. S 1** The immunofluorescence diagrams of Pax7, MyoD, Mrf4, and Myogenin in white muscle of common carp.

FITC: Target proteins incubated only with antibodies; (DAPI): Nucleus stained with DAPI; (Merge): Merge (FITC) and (DAPI). EGCG was supplemented 0, 0.05, 0.25, 0.5, or 1 g/kg.

**
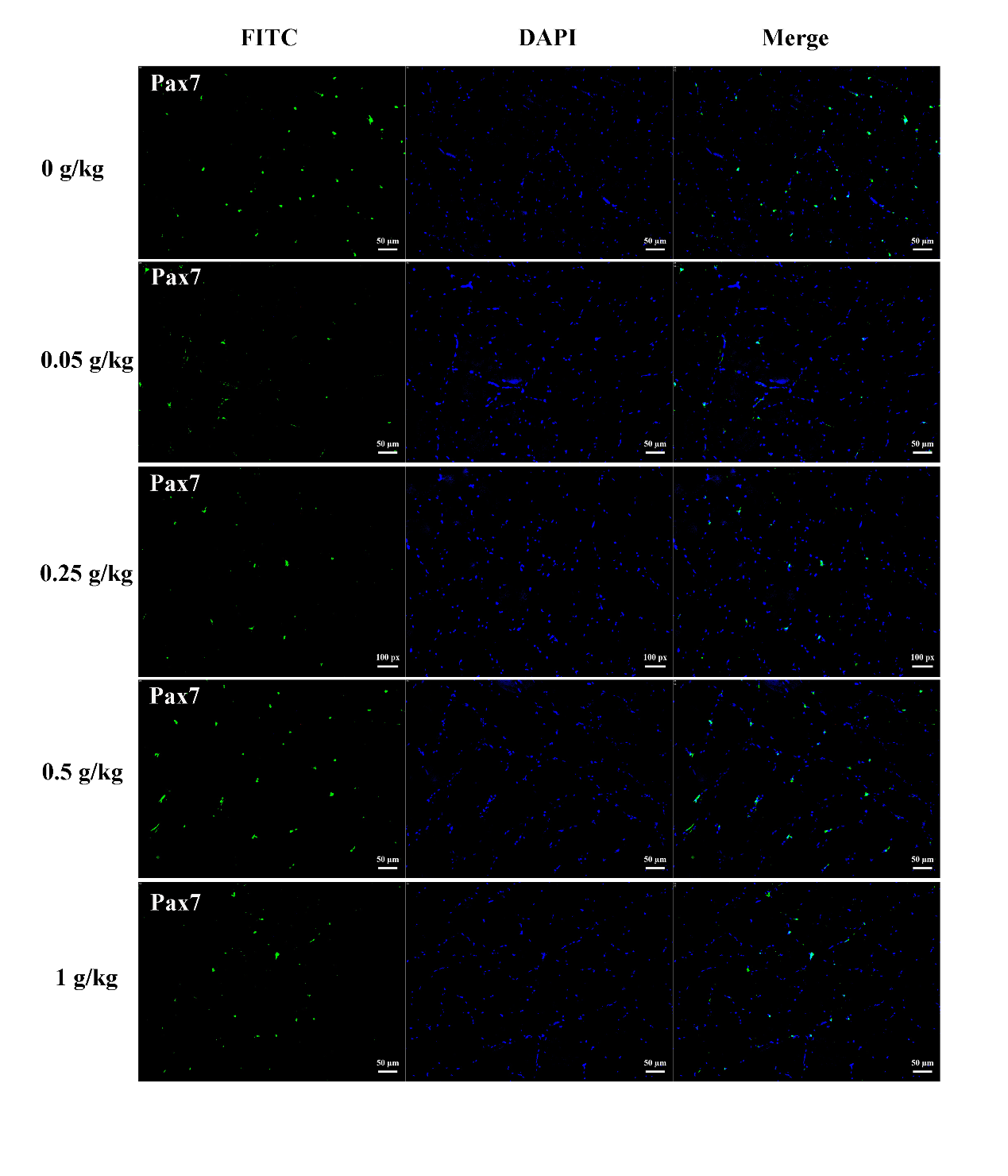
**
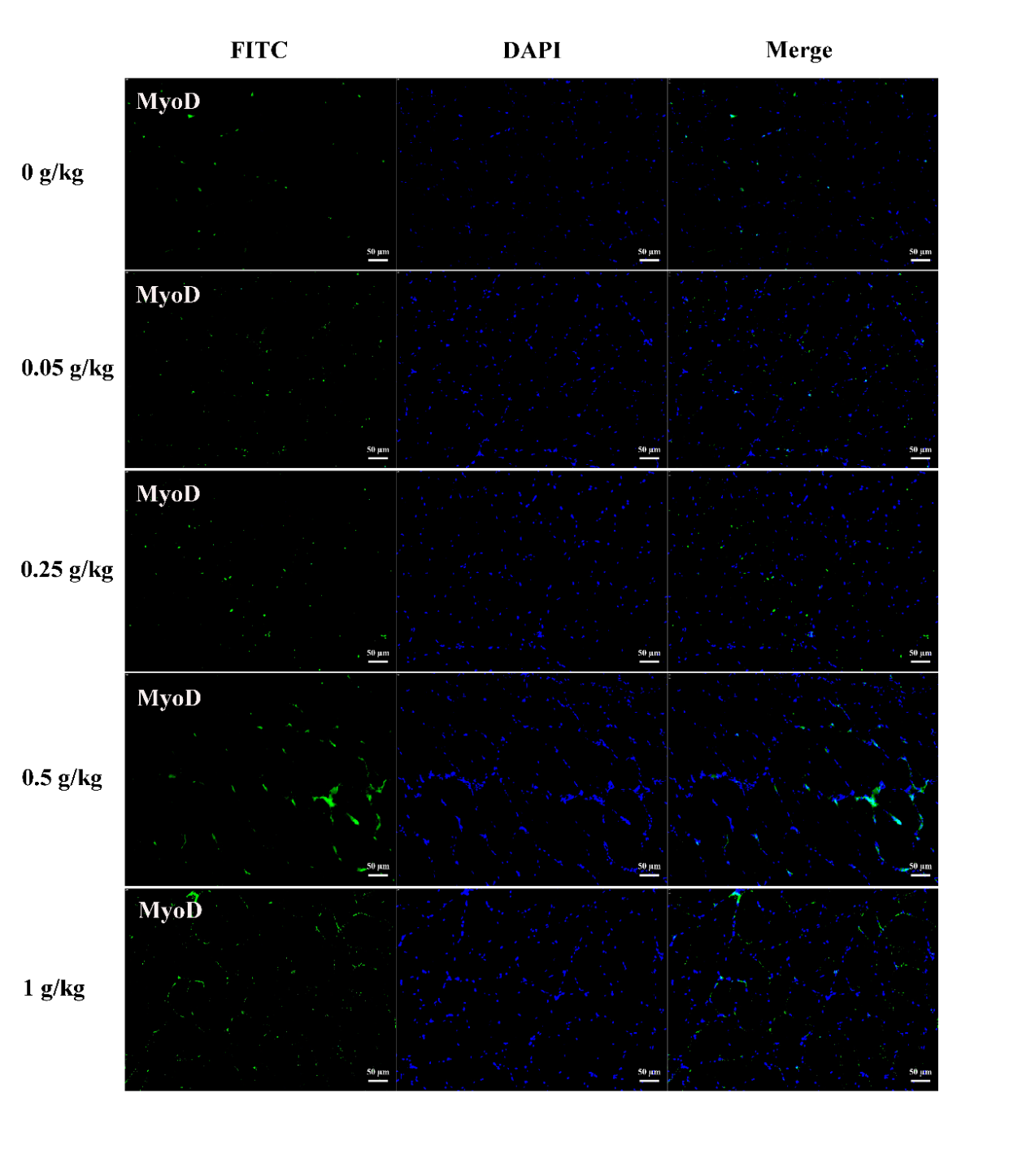


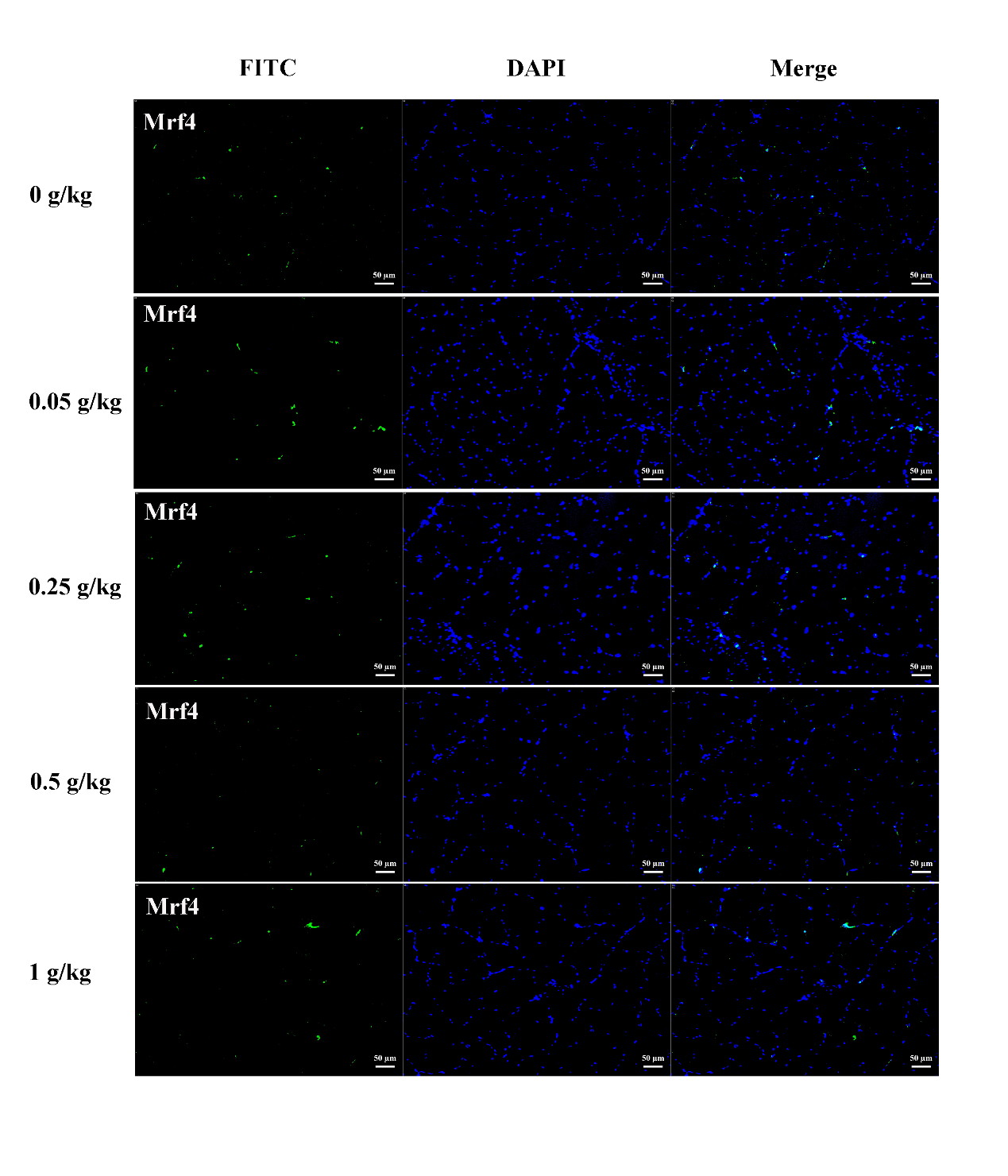


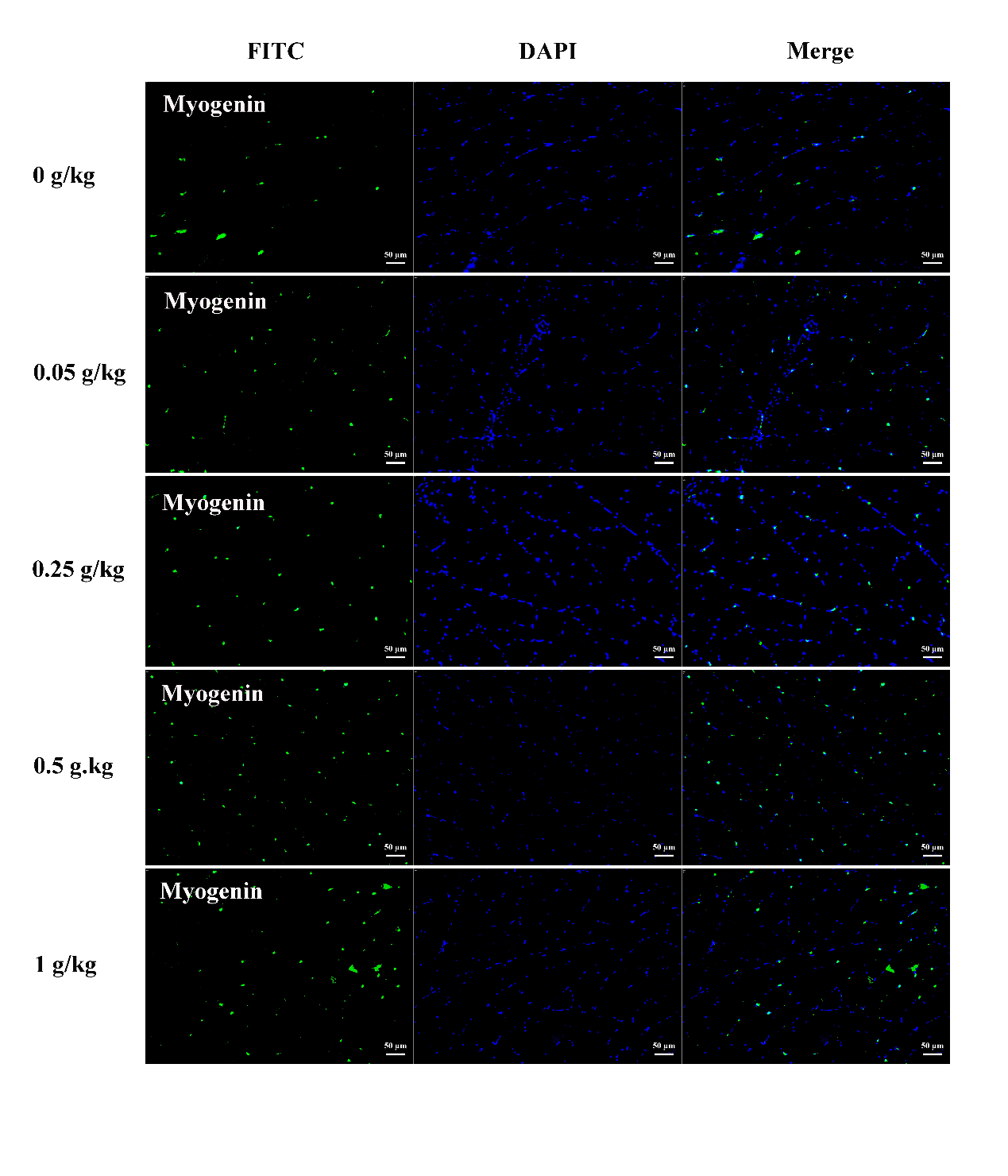

Supplement: Supplementary 3 — Immunofluorescence diagrams of Pax7, MyoD, Mrf4, and Myogenin in the white muscle of common carp. [file 7134404.f3.docx]
