# Supplementary material for: (-)-Epigallocatechin-3-O-Gallate Regulates Muscle Growth, Antioxidant Status, and Nutritional Composition of Juvenile Common Carp (Cyprinus carpio L.)
Source: Aquac Nutr. 2024 Mar 20;2024:7134404. doi: 10.1155/2024/7134404 (PMC10977338; doi:10.1155/2024/7134404)
Supplement: Supplementary 4 — Effect of dietary EGCG on the amino acid composition of white muscle of common carp (C. carpio L.) (g/kg, dry matter). [file 7134404.f4.docx]

**Table S3. Effect of dietary EGCG on amino acid composition of white muscle of common carp (*Cyprinus carpio* L*.*) (g/kg, dry matter).**

| **Index** | **Dietary EGCG levels (g/kg)** | | | | | ***P*-value** | | |
| --- | --- | --- | --- | --- | --- | --- | --- | --- |
|  | **0** | **0.05** | **0.25** | **0.5** | **1** | **A** | **L** | **Q** |
| Val | 1.73 ± 0.04 ^b^ | 2.18 ± 0.33 ^ab^ | 2.36 ± 0.22 ^a^ | 2.09 ± 0.03 ^ab^ | 1.81 ± 0.05 ^ab^ | 0.013 | 0.913 | 0.018 |
| Ile | 1.62 ± 0.04 ^c^ | 2.10 ± 0.04 ^a^ | 2.04 ± 0.02 ^ab^ | 1.98 ± 0.03 ^b^ | 1.70 ± 0.05 ^c^ | < 0.001 | 0.666 | < 0.001 |
| Leu | 3.17 ± 0.08 ^b^ | 3.96 ± 0.15 ^a^ | 3.74 ± 0.16 ^a^ | 3.88 ± 0.06 ^a^ | 3.34 ± 0.09 ^b^ | 0.002 | 0.450 | 0.001 |
| Met | 1.29 ± 0.03 ^b^ | 1.54± 0.05 ^a^ | 1.53 ± 0.01 ^a^ | 1.55 ± 0.02 ^a^ | 1.34 ± 0.01 ^b^ | 0.002 | 0.477 | < 0.001 |
| Thr | 1.82 ± 0.03 ^b^ | 1.97 ± 0.12 ^ab^ | 1.96 ± 0.03 ^ab^ | 2.04 ± 0.02 ^a^ | 1.83 ± 0.03 ^b^ | 0.016 | 0.696 | 0.016 |
| Phe | 3.23 ± 0.08 ^b^ | 2.37 ± 0.08 ^c^ | 3.59 ± 0.16 ^a^ | 3.63 ± 0.05 ^a^ | 3.39 ± 0.08 ^ab^ | < 0.001 | < 0.001 | 0.855 |
| Lys | 3.53 ± 0.10 ^b^ | 4.53 ± 0.33 ^a^ | 4.59 ± 0.03 ^a^ | 4.47 ± 0.08 ^a^ | 3.79± 0.14 ^b^ | 0.004 | 0.422 | < 0.001 |
| EAA^1^ | 16.40 ± 0.39 ^b^ | 18.65 ± 0.99 ^a^ | 19.81 ± 0.18 ^a^ | 19.64 ± 0.23 ^a^ | 17.20 ± 0.41 ^b^ | 0.004 | 0.152 | < 0.001 |
| His | 1.60 ± 0.03 ^d^ | 2.16 ± 0.04 ^b^ | 2.38 ± 0.05 ^a^ | 1.96 ± 0.06 ^c^ | 1.66 ± 0.07 ^d^ | < 0.001 | 0.595 | < 0.001 |
| Arg | 2.32 ± 0.13 ^b^ | 3.38 ± 0.05 ^a^ | 3.17 ± 0.08 ^a^ | 3.18 ± 0.03 ^a^ | 2.52 ± 0.04 ^b^ | < 0.001 | 0.420 | < 0.001 |
| SEAA ^2^ | 3.92 ± 0.16 ^b^ | 5.16 ± 0.20 ^a^ | 5.55 ± 0.11 ^a^ | 5.14 ± 0.08 ^a^ | 4.18 ± 0.10 ^b^ | < 0.001 | 0.278 | < 0.001 |
| Asp | 4.36 ± 0.09 ^b^ | 4.85 ± 0.37 ^ab^ | 4.88 ± 0.03 ^ab^ | 4.99 ± 0.08 ^a^ | 4.43 ± 0.10 ^ab^ | 0.023 | 0.628 | 0.014 |
| Glu | 6.20 ± 0.11 ^c^ | 7.36 ± 0.08 ^a^ | 7.02 ± 0.06 ^b^ | 7.17 ± 0.10 ^ab^ | 6.34 ± 0.11 ^c^ | < 0.001 | 0.786 | < 0.001 |
| Gly | 1.53 ± 0.06 ^c^ | 2.03 ± 0.04 ^a^ | 1.88 ± 0.02 ^b^ | 1.86 ± 0.04 ^b^ | 1.62 ± 0.01 ^c^ | < 0.001 | 0.981 | < 0.001 |
| Ala | 1.75 ± 0.03 ^d^ | 2.59 ± 0.02 ^a^ | 2.30 ± 0.00 ^b^ | 2.25 ± 0.04 ^b^ | 1.84 ± 0.03 ^c^ | 0.001 | 0.089 | < 0.001 |
| Ser | 1.53 ± 0.01 ^b^ | 1.76 ± 0.10 ^a^ | 1.75 ± 0.01 ^a^ | 1.79 ± 0.03 ^a^ | 1.57 ± 0.03 ^b^ | < 0.001 | 0.496 | 0.001 |
| Tyr | 1.11 ± 0.03 ^cd^ | 1.45 ± 0.03 ^a^ | 1.18 ± 0.03 ^bc^ | 1.21 ± 0.01 ^b^ | 1.08 ± 0.01 ^d^ | < 0.001 | 0.004 | < 0.001 |
| Pro | 2.12 ± 0.13 ^a^ | 1.52 ± 0.08 ^c^ | 1.73 ± 0.07 ^bc^ | 2.03 ± 0.04 ^ab^ | 1.95 ± 0.15 ^ab^ | 0.012 | 0.593 | 0.014 |
| NEAA ^3^ | 18.60± 0.32 ^b^ | 21.56 ± 0.62 ^a^ | 20.74 ± 0.07 ^a^ | 21.30 ± 0.31 ^a^ | 18.84 ± 0.11 ^b^ | < 0.001 | 0.854 | < 0.001 |
| FAA ^4^ | 15.38 ± 0.28 ^b^ | 18.60 ± 0.57 ^a^ | 17.83 ± 0.11 ^a^ | 18.06 ± 0.28 ^a^ | 15.81± 0.24 ^b^ | < 0.001 | 0.770 | < 0.001 |
| Σ TAA ^5^ | 38.85 ± 0.81 ^b^ | 45.37 ± 1.68 ^a^ | 46.10± 0.18 ^a^ | 46.07 ± 0.60 ^a^ | 40.22 ± 0.59 ^b^ | < 0.001 | 0.283 | < 0.001 |

Values are means ± SEM (n = 3). One-way ANOVA followed by Duncan’s test were used to analyze the discrepancy among all the groups. Values in the same row with different superscripts represent statistically significant difference (*P* < 0.05). The *P*-values indicate a significantly linear or quadratic dose response relationship (*P* < 0.05). Liner trend and quadratic trend were analyzed by polynomial contrasts (A, ANOVA; L, linear; Q, quadratic). EGCG was supplemented at 0, 0.05, 0.25, 0.5, or 1 g/kg. ^1^ Σ EAA: Essential amino acids; ^2^ Σ SEAA: Semi-essential amino acids; ^3^ Σ NEAA: Non-essential amino acids; ^4^ Σ FAA: Total flavor amino acids; Σ FAA=Glu + Asp + Gly + Ala + Ser;^5^ Σ TAA: Total amino acids.
