# Supplementary material for: (-)-Epigallocatechin-3-O-Gallate Regulates Muscle Growth, Antioxidant Status, and Nutritional Composition of Juvenile Common Carp (Cyprinus carpio L.)
Source: Aquac Nutr. 2024 Mar 20;2024:7134404. doi: 10.1155/2024/7134404 (PMC10977338; doi:10.1155/2024/7134404)
Supplement: Supplementary 5 — Effect of dietary EGCG on the fatty acid content in the muscle of common carp (C. carpio L.) (% total fatty acid of dry matter). [file 7134404.f5.docx]

**Table S4. Effect of dietary EGCG on fatty acid content in common carp muscle (*Cyprinus carpio* L.) (% total fatty acid of dry matter).**

| **Index** | **Dietary EGCG levels (g/kg)** | | | |  | ***P-*value** | | |
| --- | --- | --- | --- | --- | --- | --- | --- | --- |
|  | **0** | **0.05** | **0.25** | **0.5** | **1** | **A** | **L** | **Q** |
| C 14:0 | 0.48 ± 0.03 ^b^ | 0.54 ± 0.03 ^ab^ | 0.48 ± 0.01 ^b^ | 0.67 ± 0.11 ^ab^ | 0.73 ± 0.02 ^a^ | 0.002 | 0.004 | 0.244 |
| C 16:0 | 22.20 ± 0.54 ^a^ | 20.64 ± 0.07 ^bc^ | 19.86 ± 0.09 ^c^ | 21.77 ± 0.56 ^ab^ | 22.42 ± 0.39 ^a^ | 0.006 | 0.241 | 0.001 |
| C 18:0 | 7.48 ± 0.14 ^a^ | 6.53± 0.07 ^c^ | 6.57 ± 0.05 ^c^ | 7.13 ± 0.06 ^b^ | 6.99 ± 0.03 ^b^ | < 0.001 | 0.160 | < 0.001 |
| C 20:0 | 0.21 ± 0.01 ^a^ | 0.18 ± 0.01 ^b^ | 0.19 ± 0.01 ^ab^ | 0.19 ± 0.02 ^ab^ | 0.18 ± 0.00 ^b^ | 0.092 | 0.080 | 0.393 |
| SFA ^1^ | 30.37 ± 0.65 ^a^ | 27.89 ± 0.06 ^b^ | 27.09 ± 0.12 ^b^ | 29.75 ± 0.65 ^a^ | 30.31 ± 0.37 ^a^ | 0.005 | 0.369 | < 0.001 |
| C 16:1 | 2.31 ± 0.07 ^a^ | 2.55 ± 0.18 ^a^ | 2.13 ± 0.11 ^ab^ | 1.49 ± 0.46 ^b^ | 1.08 ± 0.87 ^b^ | 0.209 | 0.006 | 0.239 |
| C 18:1 | 29.63 ± 0.62 ^ab^ | 30.21± 0.65 ^a^ | 29.11 ± 0.27 ^ab^ | 27.84 ± 0.42 ^b^ | 28.58± 0.72 ^ab^ | 0.096 | 0.031 | 0.941 |
| C 20:1 | 1.52 ± 0.01 ^a^ | 1.47 ± 0.06 ^ab^ | 1.40 ± 0.01 ^ab^ | 1.41 ± 0.06 ^ab^ | 1.38 ± 0.01 ^b^ | 0.125 | 0.017 | 0.364 |
| MUFA^2^ | 33.47 ± 0.70 ^ab^ | 34.23± 0.89 ^a^ | 32.64 ± 0.28 ^ab^ | 30.73 ± 0.95 ^b^ | 31.04 ± 1.13 ^b^ | 0.060 | 0.010 | 0.709 |
| C 20:2 | 0.99 ± 0.04 | 0.99 ± 0.02 | 0.99 ± 0.02 | 1.03 ± 0.04 | 0.93 ± 0.02 | 0.303 | 0.362 | 0.160 |
| C 18:2n6 | 22.41 ± 0.61 ^c^ | 23.96 ± 0.38 ^b^ | 25.49 ± 0.23 ^a^ | 24.01 ± 0.10 ^b^ | 23.93 ± 0.27 ^b^ | 0.002 | 0.031 | 0.001 |
| C 18:3n6 | 0.52 ± 0.02 ^b^ | 0.57 ± 0.01 ^ab^ | 0.65 ± 0.03 ^a^ | 0.51 ± 0.01 ^b^ | 0.52 ± 0.05 ^b^ | 0.020 | 0.539 | 0.012 |
| C 20:3n6 | 2.21 ± 0.12 | 2.19 ± 0.06 | 2.33 ± 0.07 | 2.40 ± 0.05 | 2.20 ± 0.12 | 0.406 | 0.506 | 0.221 |
| n-6 PUFA^3^ | 25.13 ± 0.73 ^c^ | 26.73 ± 0.43 ^b^ | 28.47 ± 0.21 ^a^ | 26.91 ± 0.15 ^b^ | 26.55 ± 0.43 ^b^ | 0.005 | 0.056 | 0.001 |
| C 18:3n3 | 0.61 ± 0.04 ^c^ | 0.77 ± 0.01 ^b^ | 0.85 ± 0.02 ^a^ | 0.66 ± 0.01 ^c^ | 0.65 ± 0.00 ^c^ | < 0.001 | 0.663 | < 0.001 |
| C 20:3n3 | 4.65 ± 0.26 ^ab^ | 4.46 ± 0.24 ^b^ | 4.87 ± 0.12 ^ab^ | 5.28 ± 0.09 ^a^ | 5.03 ± 0.22 ^ab^ | 0.095 | 0.029 | 0.887 |
| C 20:5n3 (EPA) | 0.60 ± 0.01 ^ab^ | 0.51 ± 0.02 ^b^ | 0.49 ± 0.03 ^b^ | 0.72 ± 0.10 ^a^ | 0.76 ± 0.06 ^a^ | 0.017 | 0.012 | 0.026 |
| C 22:6n3  (DHA) | 4.18 ± 0.25 ^b^ | 4.42 ± 0.15 ^ab^ | 4.62 ± 0.07 ^ab^ | 4.92 ± 0.11 ^a^ | 4.73 ± 0.19 ^ab^ | 0.074 | 0.012 | 0.258 |
| n-3 PUFA^4^ | 10.04 ± 0.54 ^b^ | 10.16 ± 0.42 ^b^ | 10.82 ± 0.19 ^ab^ | 11.58 ± 0.24 ^a^ | 11.17 ± 0.48 ^ab^ | 0.090 | 0.015 | 0.537 |
| PUFA^5^ | 36.16 ± 1.26 ^b^ | 37.88 ± 0.87 ^ab^ | 40.27 ± 0.22 ^a^ | 39.52 ± 0.36 ^a^ | 38.65 ± 0.92 ^ab^ | 0.041 | 0.029 | 0.022 |
| UFA ^6^ | 69.63 ± 0.65 ^b^ | 72.11 ± 0.06 ^a^ | 72.91 ± 0.12 ^a^ | 70.25 ± 0.65 ^b^ | 69.69 ± 0.37 ^b^ | 0.005 | 0.242 | < 0.001 |
| n-3/n-6 | 0.40 ± 0.01 ^ab^ | 0.38 ± 0.01 ^b^ | 0.38 ± 0.01 ^b^ | 0.43 ± 0.01 ^a^ | 0.42 ± 0.01 ^a^ | 0.016 | 0.016 | 0.099 |
| HUFA^7^ | 11.64 ± 0.63 ^b^ | 11.58 ± 0.47 ^b^ | 12.30 ± 0.27 ^ab^ | 13.32 ± 0.26 ^a^ | 12.72 ± 0.59 ^ab^ | 0.114 | 0.025 | 0.675 |
| IA^8^ | 0.35 ± 0.01 ^a^ | 0.32 ± 0.00 ^b^ | 0.30 ± 0.00 ^b^ | 0.35 ± 0.02 ^a^ | 0.36 ± 0.01 ^a^ | 0.004 | 0.048 | 0.001 |
| IT ^9^ | 0.51 ± 0.02 ^a^ | 0.45 ± 0.01 ^bc^ | 0.42 ± 0.00 ^c^ | 0.46 ± 0.01 ^bc^ | 0.48 ± 0.01 ^ab^ | 0.010 | 0.338 | 0.001 |
| HPI ^10^ | 2.89 ± 0.09 ^b^ | 3.16 ± 0.03 ^a^ | 3.35 ± 0.01 ^a^ | 2.89 ± 0.13 ^b^ | 2.75 ± 0.06 ^b^ | 0.002 | 0.043 | < 0.001 |
| LA/ALA^11^ | 37.03 ± 1.23 ^a^ | 31.16 ± 0.11 ^b^ | 30.11 ± 0.54 ^b^ | 36.18 ± 0.63 ^a^ | 36.90 ± 0.16 ^a^ | < 0.001 | 0.049 | < 0.001 |

Values are means ± SEM (n = 3). One-way ANOVA followed by Duncan’s test were used to analyze the discrepancy among all the groups. Values in the same row with different superscripts represent statistically significant difference (*P* < 0.05). The *P*-values indicate a significantly linear or quadratic dose response relationship (*P* < 0.05). Liner trend and quadratic trend were analyzed by polynomial contrasts (A, ANOVA; L, linear; Q, quadratic). EGCG was supplemented at 0, 0.05, 0.25, 0.5, or 1 g/kg. ^1^ Σ SFA, Saturated fatty acid; ^2^ Σ MUFA, Monounsaturated fatty acid; ^3^ Σn-6 PUFA, n-6 polyunsaturated fatty acids; ^4^ Σ n-3 PUFA, n-3 polyunsaturated fatty acids; ^5^ Σ PUFA, polyunsaturated fatty acids; ^6^ UFA, Unsaturated fatty acid; ^7^ Σ HUFA, Highly Unsaturated Fatty Acid; ^8^ IA, index of atherogenicity. IA = [C 12:0 + (4 × C 14:0) + C 16:0] / UFA; ^9^ IT, index of thrombogenicity. IT = (C 14:0 + C 16:0 + C 18:0) / [(0.5 × MUFA) + (0.5 × n-6 PUFA) + (3 × n-3 PUFA) + (n-3/n-6)]; ^10^ HPI, health-promoting index. HPI = UFA / [C 12:0 + (4 × C 14:0) + C 16:0]; ^11^ LA/ALA = C 18:2n-6 /C 18:3.
